# Supplementary material for: Thousands of previously unknown phages discovered in whole-community human gut metagenomes
Source: Microbiome. 2021 Mar 29;9:78. doi: 10.1186/s40168-021-01017-w (PMC8008677; doi:10.1186/s40168-021-01017-w)
Supplement: Supplementary file 4 — Additional file 3. Distribution of the marker profiles identified on each phage genome and pie chart representation of the gut phage taxonomy. (A) Histogram of marker proteins detected on each phage genome recovered from human gut metagenomes. Abbreviations are as follows: M, major capsid protein; P, portal, T, terminase large subunit. (B) Taxonomic assignments of the dereplicated contigs (n = 1,886), with the outermost ring corresponding to ICTV families. [file 40168_2021_1017_MOESM4_ESM.pdf]

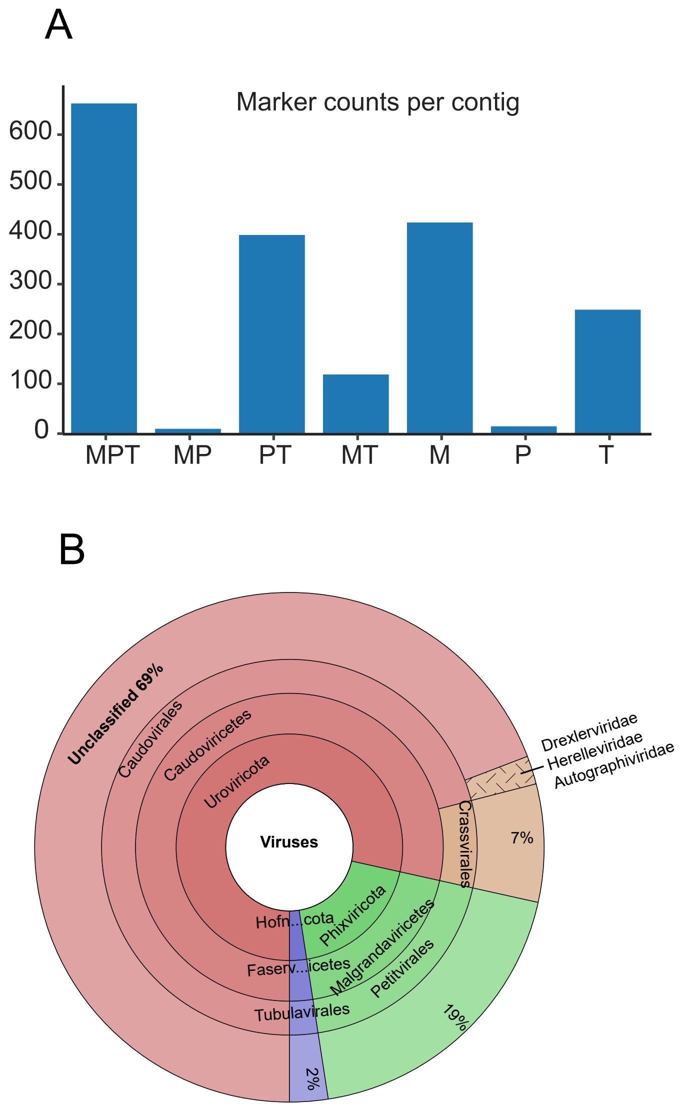

Distribution of the marker profiles identified on each phage genome and pie chart representation of the gut phage taxonomy. (A) Histogram of marker proteins detected on each phage genome recovered from human gut metagenomes. Abbreviations are as follows: M, major capsid protein; P, portal, T, terminase large subunit. (B) Taxonomic assignments of the dereplicated contigs (n = 1,886), with the outermost ring corresponding to ICTV families.
