# Supplementary material for: Thousands of previously unknown phages discovered in whole-community human gut metagenomes
Source: Microbiome. 2021 Mar 29;9:78. doi: 10.1186/s40168-021-01017-w (PMC8008677; doi:10.1186/s40168-021-01017-w)
Supplement: Supplementary file 7 — Additional file 6. Genome maps of predicted anti-CRISPR proteins (Acrs) in uncharacterized Bifidobacteria phages. Open reading frames are colored according to function: large terminase subunit (red), structural components (blue), replication (orange), integrase (pink), general function (green) and unknown (grey). The candidate Acrs are indicated with a dashed box. [file 40168_2021_1017_MOESM7_ESM.pdf]

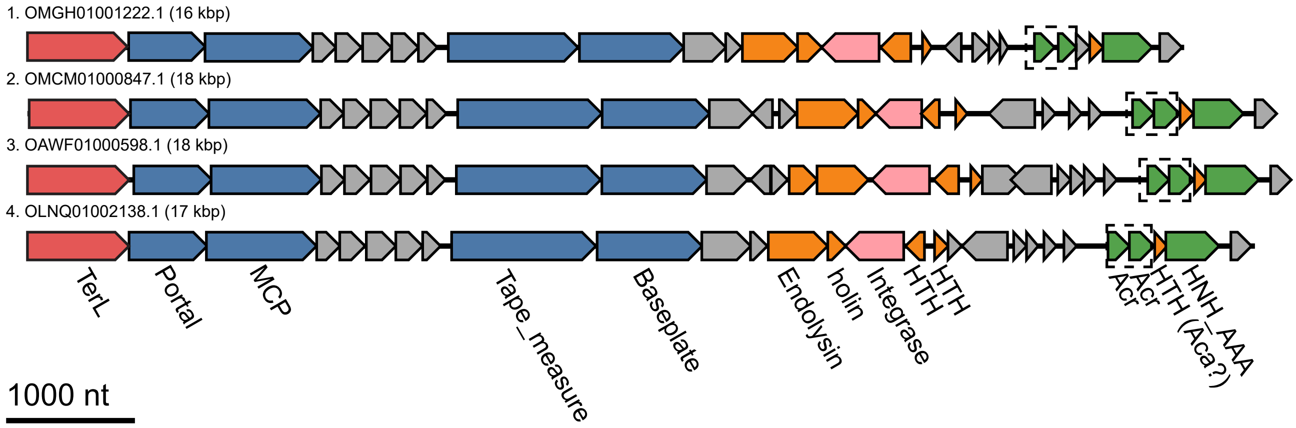


**Genome maps of predicted anti-CRISPR proteins (Acrs) in uncharacterized Bifidobacteria phages.** Open reading frames are colored according to function: large terminase subunit (red), structural components (blue), replication (orange), integrase (pink), general function (green) and unknown (grey). The candidate Acrs are indicated with a dashed box.
