# Supplementary material for: Thousands of previously unknown phages discovered in whole-community human gut metagenomes
Source: Microbiome. 2021 Mar 29;9:78. doi: 10.1186/s40168-021-01017-w (PMC8008677; doi:10.1186/s40168-021-01017-w)
Supplement: Supplementary file 9 — Additional file 8. Phylogenetic tree of the MCP, primase and portal proteins encoded by “Quimbyviridae” phages. [file 40168_2021_1017_MOESM9_ESM.pdf]

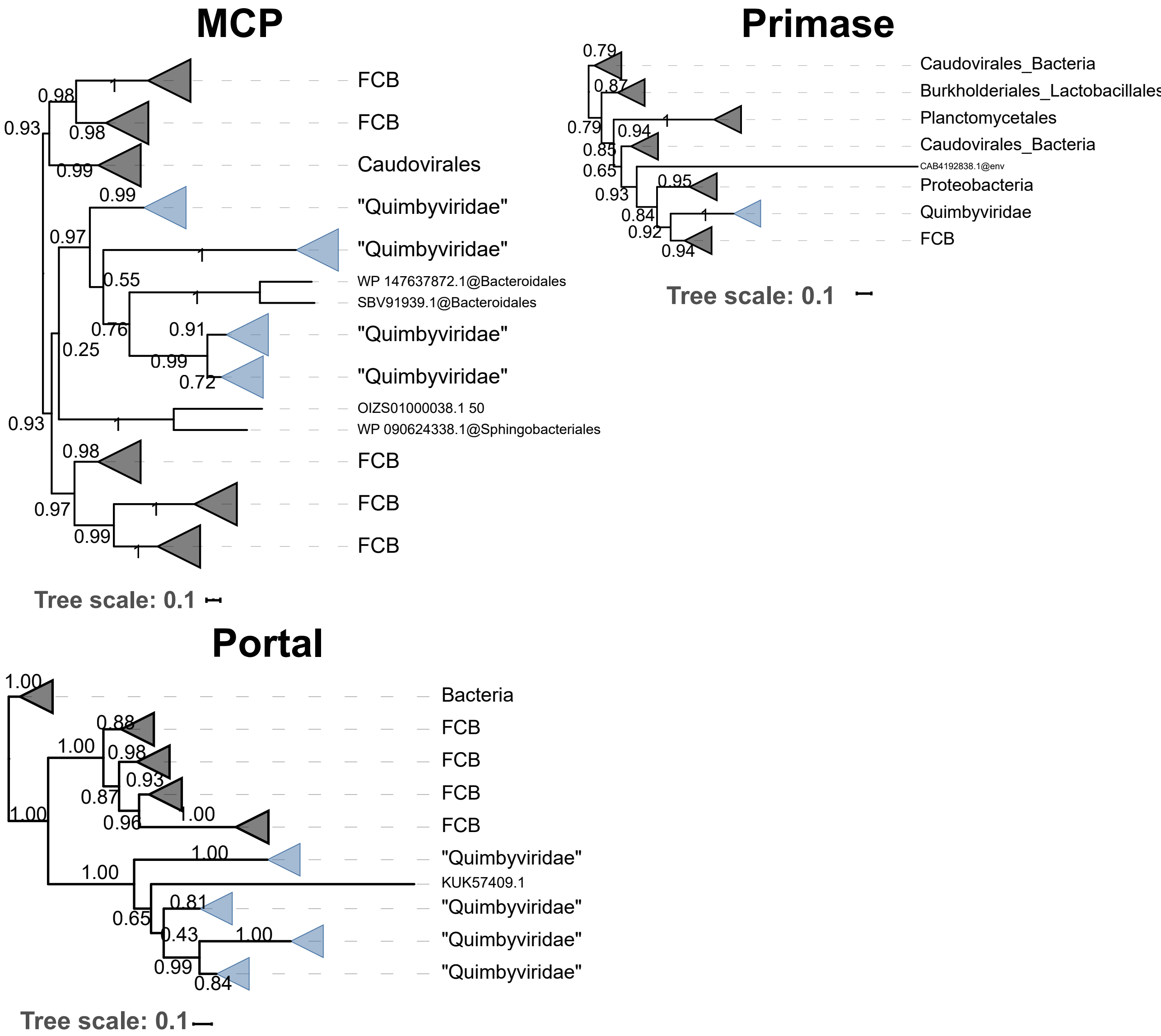

Phylogenetic tree of the MCP, primase and portal proteins encoded by "Quimbyviridae" phages
