# Supplementary material for: Thousands of previously unknown phages discovered in whole-community human gut metagenomes
Source: Microbiome. 2021 Mar 29;9:78. doi: 10.1186/s40168-021-01017-w (PMC8008677; doi:10.1186/s40168-021-01017-w)
Supplement: Supplementary file 11 — Additional file 10. HHPred alignments of four Quimbyviridae proteins and one Gratiaviridae protein with their top-scoring templates, including a replication initiator protein, a cytosine-specific methyltransferase, an adenine-specific methyltransferase, a MutY nuclease and HipA kinase. [file 40168_2021_1017_MOESM11_ESM.docx]

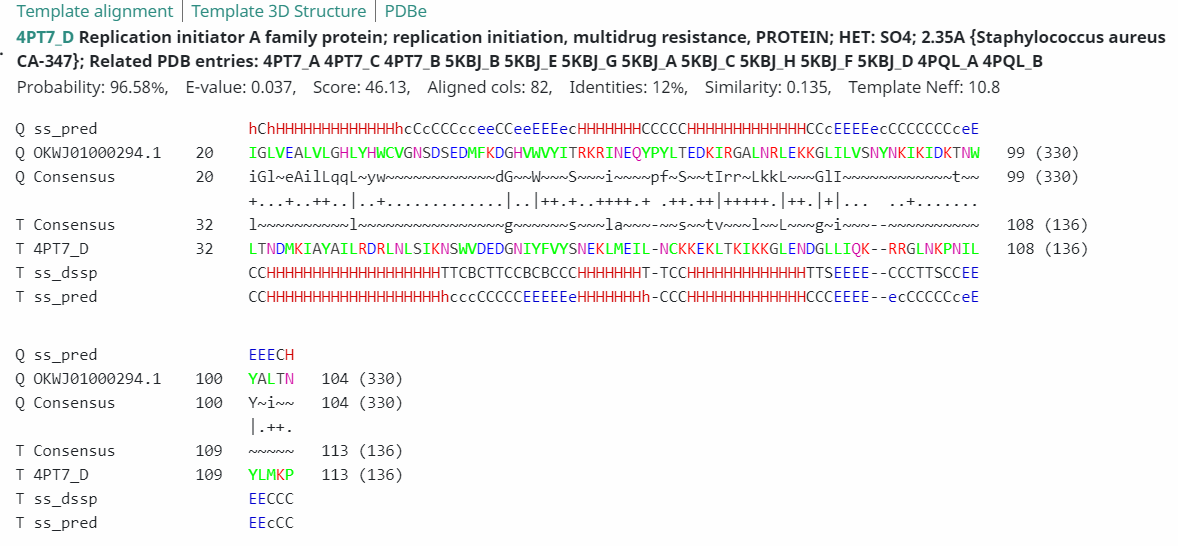


HHPred alignment of a Quimbyviridae protein (OKWJ01000294.1_36) with the N-terminal domain of RepA (PDB 4PT7_D)


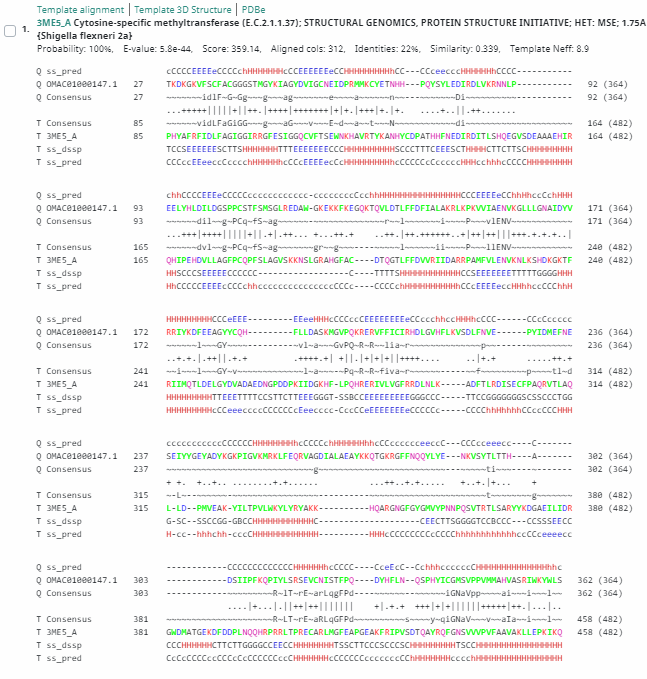


HHPred alignment of a Quimbyviridae protein (OMAC01000147.1_43) with a cytosine-specific methyltransferase (PDB 3ME5_A)


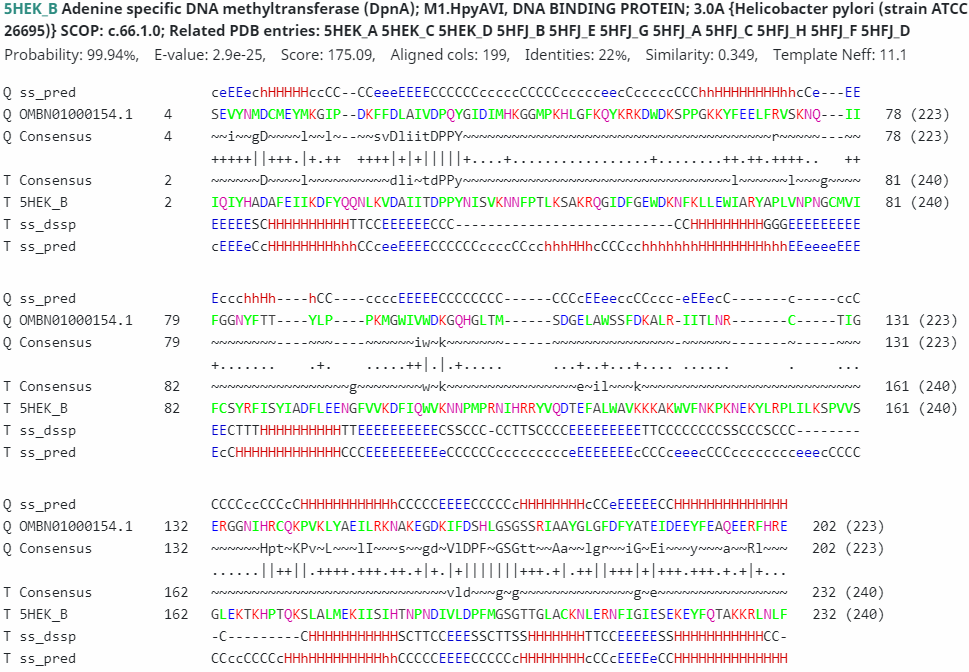


HHPred alignment of a Quimbyviridae protein (OMBN01000154.1_53) with an adenine-specific methyltransferase (PDB 3ME5_A)


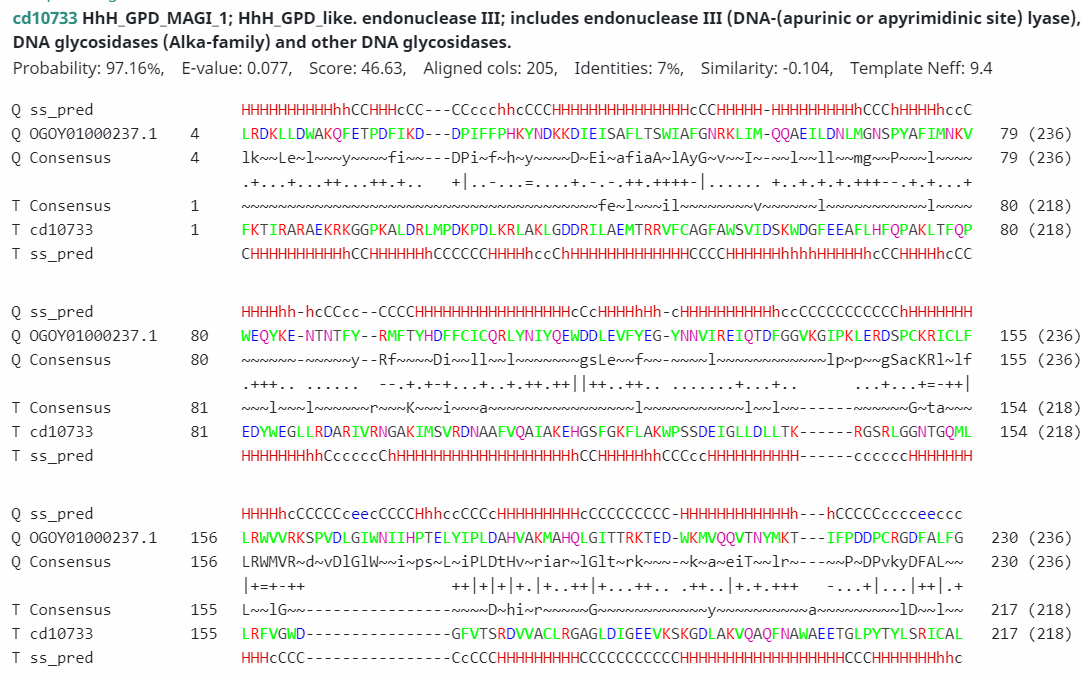


HHPred alignment of a Quimbyviridae protein (OGOY01000237.1_47) with a MutY-like nuclease (PDB cd10733)


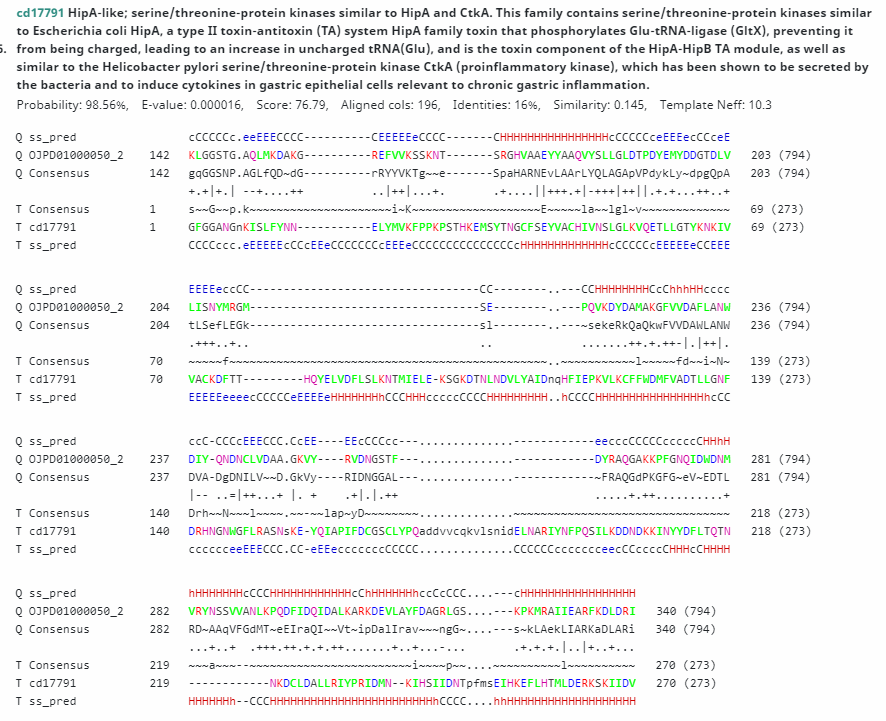


HHPred alignment of a Gratiaviridae protein (OJPW01000080.1_28) with a HipA-like kinase (CDD17791).
