# Supplementary material for: Thousands of previously unknown phages discovered in whole-community human gut metagenomes
Source: Microbiome. 2021 Mar 29;9:78. doi: 10.1186/s40168-021-01017-w (PMC8008677; doi:10.1186/s40168-021-01017-w)
Supplement: Supplementary file 12 — Additional file 11. Phylogenetic tree of the polA and dnaG genes in Flanders-like phages. Branches composed of GenBank phages are colored in orange and branches of gut metagenomic phages in blue. Branches with sequences labelled as bacteria in the GenBank database, likely representing cryptic prophages, are colored in grey. [file 40168_2021_1017_MOESM12_ESM.pdf]

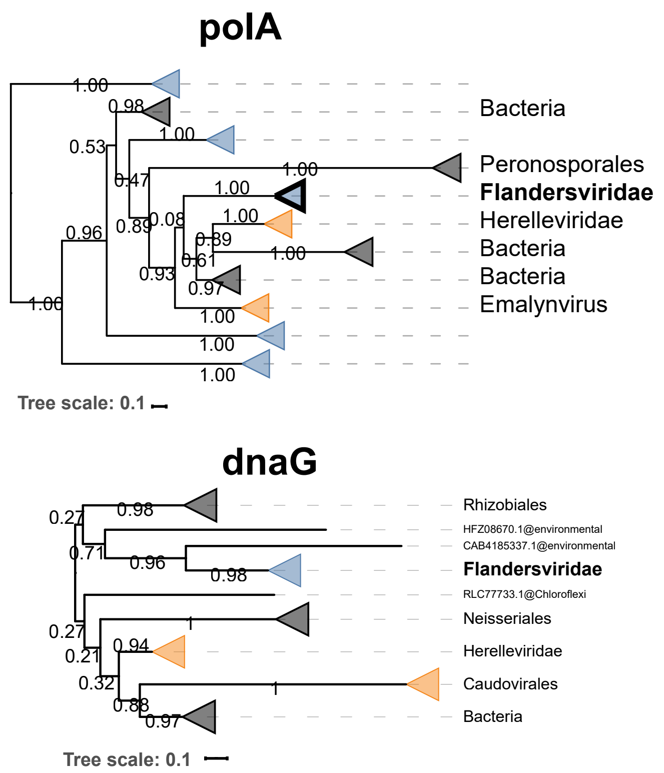

Phylogenetic tree of the polA and dnaG genes in Flanders-like phages. Branches composed of GenBank phages are colored in orange and branches of gut metagenomic phages in blue. Branches with sequences labelled as bacteria in the GenBank database, likely representing cryptic prophages, are colored in grey
