# Supplementary material for: Thousands of previously unknown phages discovered in whole-community human gut metagenomes
Source: Microbiome. 2021 Mar 29;9:78. doi: 10.1186/s40168-021-01017-w (PMC8008677; doi:10.1186/s40168-021-01017-w)
Supplement: Supplementary file 13 — Additional file 12. Coverage heatmap of a “Flandersviridae” genome across human gut viromes. The coverage of the most abundant “Flandersviridae” phage genome (accession OLOC0100071.1) is plotted as a heatmap, scaled from 0 – 100x fold coverage per 100 bp window. [file 40168_2021_1017_MOESM13_ESM.pdf]

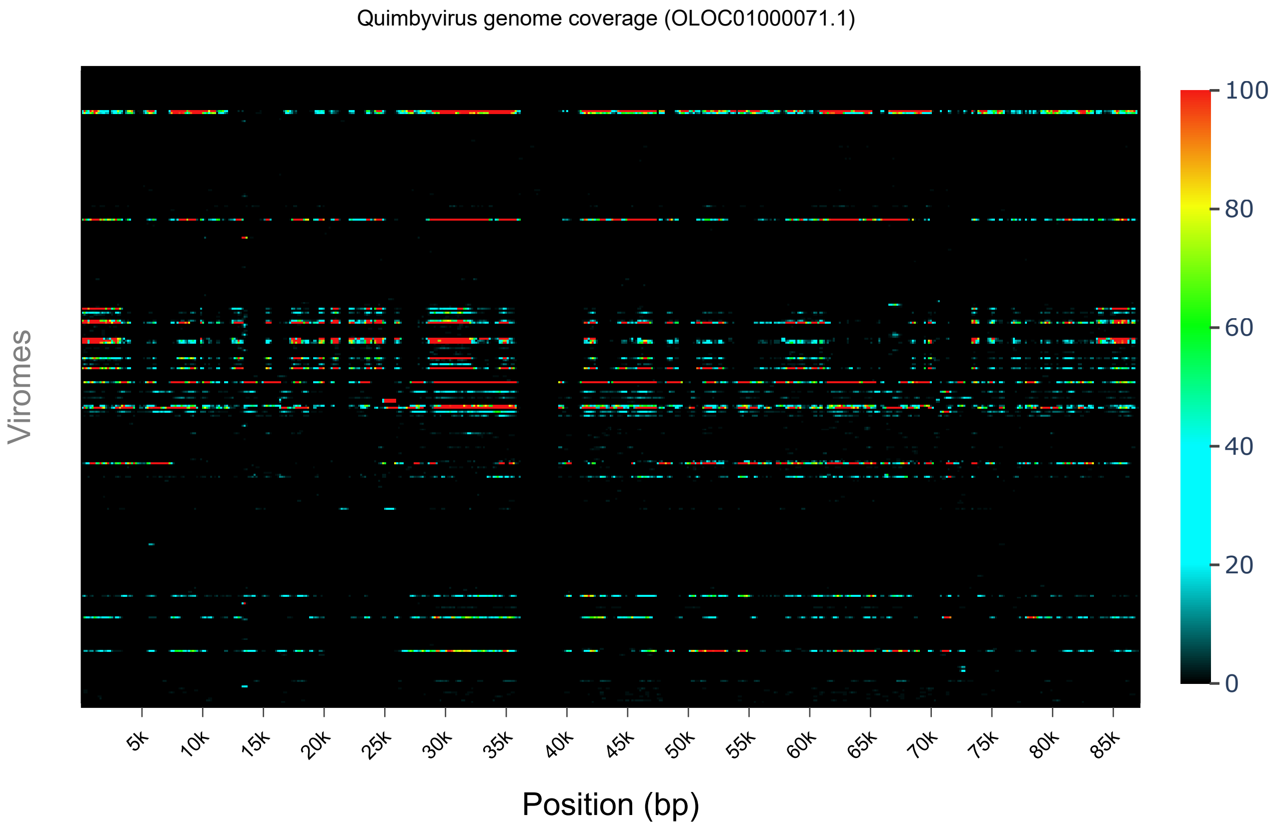

Coverage heatmap of a "Flandersviridae" genome across human gut viromes. The coverage of the most abundant "Flandersviridae" phage genome (accession OLOC0100071.1) is plotted as a heatmap, scaled from 0 – 100x fold coverage per 100 bp window.
