# Supplementary figures and images for: Thousands of previously unknown phages discovered in whole-community human gut metagenomes
Source: Microbiome. 2021 Mar 29;9:78. doi: 10.1186/s40168-021-01017-w (PMC8008677; doi:10.1186/s40168-021-01017-w)

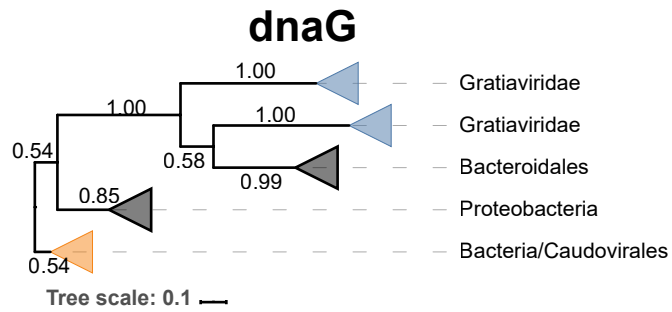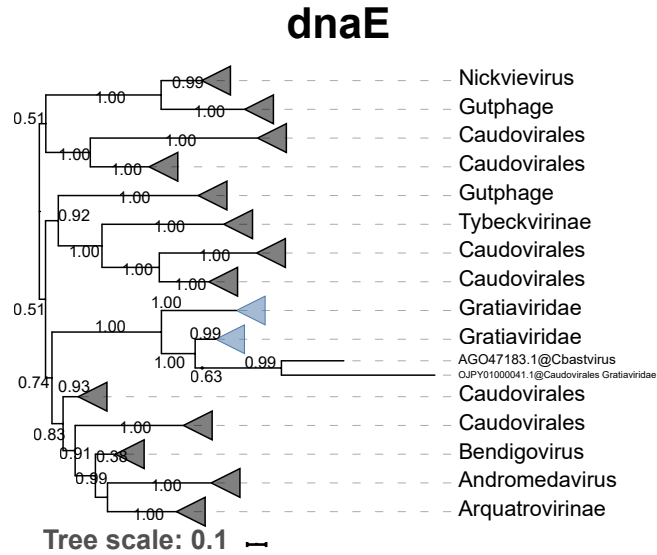

**Phylogenetic tree of the dnaG and dnaE genes in Gratiaviridae phages**

Supplement: Supplementary file 14 — Additional file 13. Phylogenetic tree of the dnaG and dnaE genes in Gratiaviridae phages. [file 40168_2021_1017_MOESM14_ESM.pdf]
