# Supplementary material for: Thousands of previously unknown phages discovered in whole-community human gut metagenomes
Source: Microbiome. 2021 Mar 29;9:78. doi: 10.1186/s40168-021-01017-w (PMC8008677; doi:10.1186/s40168-021-01017-w)
Supplement: Supplementary file 10 — Additional file 9. Alignment of the template and variable repeats from the Quimbyvirus DGRs. The template repeat (TR) from Quimbyvirus is the first listed sequence and is followed by the variable repeats from either ORF80 (VR1) or ORF 47 (VR2) encoded in phage genomes that are nearly identical to Quimbyvirus (> 95 % average nucleotide identity). A total of 21 adenine residues (green) in the template repeat exhibit a least one substitution in a corresponding variable repeat. [file 40168_2021_1017_MOESM10_ESM.pdf]

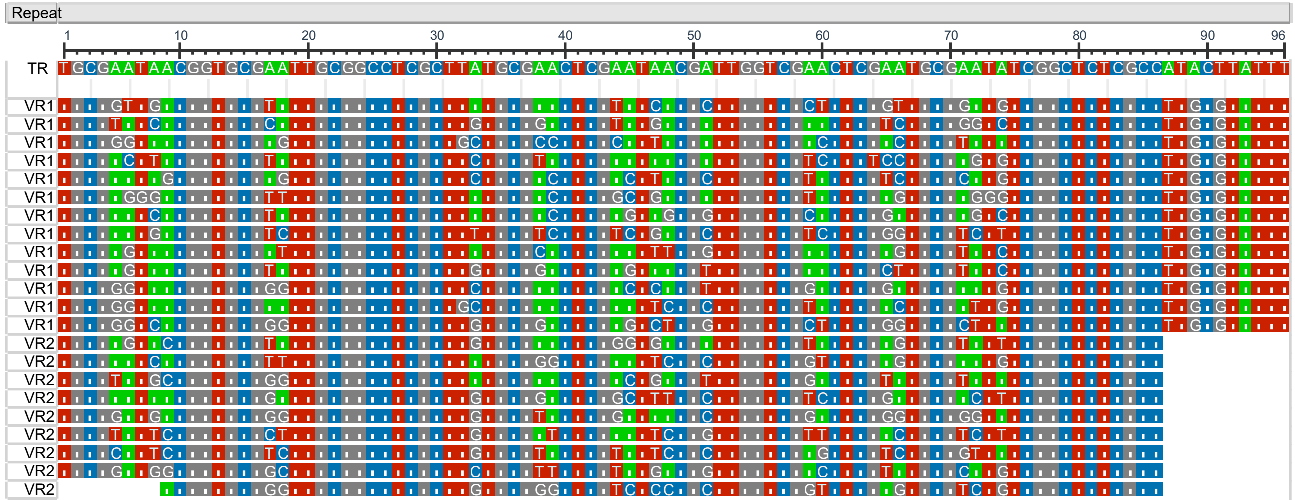

Alignment of the template and variable repeats from the Quimbyvirus DGRs. The template repeat (TR) from Quimbyvirus is the first listed sequence and is followed by the variable repeats from either ORF80 (VR1) or ORF 47 (VR2) encoded in phage genomes that are nearly identical to Quimbyvirus (> 95 % average nucleotide identity). A total of 21 adenine residues (green) in the template repeat exhibit a least one substitution in a corresponding variable repeat.
